# Evaluation of Inter-Method Reliability Between Palarum PUP Smart Socks^®^ and the Opal v1 Mobility Lab System Among Ambulatory Adults

**DOI:** 10.3390/s26185730

**Published:** 2026-09-09

**Authors:** Pamela J. Wright, Alicia Flach, Kelsie M. Bryant, Kesheng Wang

**Affiliations:** 1 Advancing Chronic Care Outcomes Through Research and iNnovation (ACORN) Center, Biobehavioral Health & Nursing Science Department, College of Nursing, University of South Carolina, Columbia, SC 29208, USA; kesheng@mailbox.sc.edu; 2 Department of Physical Therapy, College of Health Professions, Virginia Commonwealth University, Richmond, VA 23298, USA; flacha2@vcu.edu; 3 Department of Exercise Science, Arnold School of Public Health, Columbia, SC 29208, USA; kelsiemb@email.sc.edu

**Keywords:** wearable technology, validation study, reproducibility of results, early ambulation, gait analysis

## Abstract

Early mobilization (EM) can mitigate hospital-acquired deconditioning by preserving prehospital functional status. EM measurement is challenging due to concerns such as accuracy and patient comfort. Palarum PUP^®^ Smart Socks were designed for the hospital setting to track step count and time out of bed. The purpose of this study was to evaluate the inter-method reliability of the Palarum PUP^®^ Smart Socks compared with the Opal v1 Mobility Lab system. Using a cross-sectional design, participants wore Smart Socks and six mobility sensors while completing three trials of a 20 m walk at a self-selected pace. Step count, stride length, gait speed, and duration were recorded. Differences between the two measurement systems were assessed using paired *t*-tests, agreement was evaluated using Bland–Altman analyses, and reliability was assessed using intraclass correlation coefficients (ICCs) with 95% confidence intervals. Fifty-three participants were enrolled (mean age 37.9 ± 16.7 years; 64% female; 75% White; BMI 28.2 ± 5.9 kg/m^2^). ICCs demonstrated excellent reliability for step count and duration (ICC > 0.90) moderate reliability for gait speed (ICC = 0.52), and poor reliability for stride length (ICC = 0.30). Reliability was statistically significant for step count, gait speed, duration, and stride length (all *p* < 0.01). Findings support the reliability of Palarum PUP^®^ Smart Socks among ambulatory adults. By overcoming many limitations of traditional wearables in hospital settings, Smart Socks may be particularly well suited for EM programs. However, further validation is needed among hospitalized adults who may have slower gait speed and/or altered gait patterns.

## 1. Introduction

Hospital-acquired deconditioning is defined as compromised physiological or cognitive functioning manifested as reduced capability to perform activities of daily living during hospitalization due to immobility or reduced physical activity [1]. Approximately 30–50% of hospitalized adults will experience hospital-acquired deconditioning, with the risk increasing among adults aged 65 years or older [2]. Early mobilization (EM) mitigates the adverse effects of hospitalization by preserving prehospital functional status and promoting engagement, autonomy, and mental well-being [3]. EM involves initiating mobility activities once patients are medically stable, typically within 48–72 h of admission [4]. EM is also associated with reduced length of stay and hospital readmissions [5,6]. Findings from a study conducted in a cardiovascular intensive care unit revealed that EM yielded a 2.6% reduction in mortality [7], and a U.S. registry of 285,000 heart failure hospitalizations showed that hospitals with higher EM use had shorter stays and 24% lower 30-day readmission rates [8]. Despite guideline support and demonstrated benefits, EM implementation and sustainability in hospitals remain poorly studied due to challenges in measuring EM [9].

Step count is a strong predictor of health outcomes in older adults and is increasingly recognized as a meaningful hospital metric [10]. Hospitalized patients typically average fewer than 2500 steps per day, a level associated with longer lengths of stay and increased complications [10]. In contrast, achieving more than 5580 steps per day has been linked to reduced all-cause mortality [11]. Despite its clinical relevance, step count monitoring in hospital settings has been inconsistent, relying on manual observation or commercial wearable devices. These approaches are limited by issues such as reduced accuracy, device placement variability, and battery or data constraints [12].

Beyond step count, gait analysis (i.e., stride length, gait speed, and walking duration) provides additional clinically important insight, especially during rehabilitation for improved functional status and prediction of fall risk [13]. While laboratory-based optical motion capture systems are the gold standard and offer high accuracy, they are costly and impractical for real-world use [14]. Alternatively, wearable technology is of growing interest in the hospital setting as a more affordable and convenient device to assess EM [15]. However, to date, wearables have presented several challenges in hospital settings, such as measurement accuracy [16], integration into the clinical workflow [17], patient discomfort, and patient adherence [18,19].

Non-slip Palarum PUP^®^ Smart Socks (Lebanon, OH, USA) are a new innovative wearable mobility monitoring device designed for hospital use. Smart socks offer a practical and scalable solution for hospital mobility monitoring because patients must wear non-slip socks for safety. By integrating sensing capabilities into an item that patients routinely use, Smart Socks eliminate inconsistent placement and poor adherence associated with other wearable devices. Smart Socks can continuously and unobtrusively track step count and gait parameters via integrated conductive pressure sensors woven into the fabric beneath the toes and on the heel area and a Bluetooth transmitter on the upper outer edge of the socks. Additionally, the system directly integrates with the electronic health record, transmitting data in real time. Thus, the purpose of this study was to evaluate the inter-method reliability between Palarum PUP^®^ Smart Socks and the Opal v1 Mobility Lab System (Mobility Lab, APDM Inc., Portland, OR, USA).

## 2. Materials and Methods

### 2.1. Ethical Considerations

The study was conducted in accordance with the principles stated in the Declaration of Helsinki and 45 CFR 46.104(d)(2) and 45 CFR 46.111(a)(7). The University of South Carolina (USC) Institutional Review Board (IRB) approved the study (STUDY00000525; 22 December 2025). Written informed consent was obtained from all participants.

### 2.2. Study Design and Setting

A cross-sectional design was employed to evaluate inter-method reliability. Eligible participants were scheduled to meet at the USC mobility lab housed at the Arnold School of Public Health.

### 2.3. Sample Size Calculation and Participants

The power and sample size were calculated based on intra-correlation coefficient (ICC)/reliability hypothesis test [20,21]. Given ρ0 = minimally acceptable ICC under H_0_ (e.g., 0.60 or 0.70) and ρ1 = expected ICC under H_1_ (what you realistically expect—e.g., 0.80), k = number of repeated measurements per subject (3 trials → k = 3), significance level (α) = 5%, power (1 − β) = 80%, and attrition rate at 25%, the sample size was determined to be 50 participants.

Participants were recruited via flyers, a university list-serv, and social media. Inclusion criteria were an age of 18 years or older, willingness to wear Smart Socks, and capability of walking 20 m. Participants received a $25 gift card for their time and participation in the study.

### 2.4. Data Collection

Each participant was asked to remove any footwear and apply a pair of mobility Smart Socks (available in small, medium, large, and extra-large). Smart Socks (V2.0, Palarum, LLC, Lebanon, OH, USA) were affixed with Bluetooth transmitters placed on the upper outer edge of the Smart Socks. Sensor sampling frequency was 18–25 Hz. Participant identification number (ID), weight, and height were entered into the system’s tablet (Mobility, V2.2), and connection was verified when the participants stood and applied pressure to each foot. Proprietary algorithms were used to calculate each measure.

Participants also wore six body-worn inertial sensors, one located on the sternum, one on the low back, one on each wrist, and one on the dorsum of each foot. The sensors measured spatiotemporal gait parameters using the Opal v1 Mobility Lab system (Mobility Lab, APDM Inc., Portland, OR, USA), a commercially available system that allows the sensors to be wirelessly synchronized (Opal v1 System) and software (Mobility Lab, APDM Inc., Portland, OR, USA) that allows for data collection and automatic analysis via algorithms [22]. Step count was derived from foot-mounted sensors; stride length was calculated using the Mobility Lab software algorithm based on foot sensor data. Reliability has been reported as excellent with an intraclass correlation (ICC) of 0.91–0.99 [23]. Participant ID, weight, and height were entered into the computer.

For both systems, step count was the number of steps taken during each trial. Stride length was captured by measuring the distance traveled by each foot averaged over the total number of steps. Gait speed was measured as meters per second and was averaged across each lower extremity over the gait cycles captured. Walking duration was the total time it took to complete a 20 m walking trial.

The walkway for the instrumented walking tests was 10 m long. One trial involved the participant walking 10 m, turning around, and walking another 10 m, returning to the starting line for a total of 20 m. Participants stood directly behind the starting line. The APDM provided a 3 s countdown followed by a loud alarm indicating for the participants to start walking at a self-selected pace. The Palarum operator initiated the system for data collection at the sound of the alarm. When participants returned to baseline, the APDM and Palarum operators clicked stop on the respective systems. Each participant completed three trials of the 20 m walk test (20MWT) following the same procedure. Turning time was included in the data collection. The operators of each system had no roles in device configuration, data processing, or data interpretation.

### 2.5. Data Analysis

Demographic characteristics were calculated as means and standard deviations for continuous variables and percentages for categorical variables. For each participant and each gait parameter, the measurements from the three repeated walking trials were first averaged separately for each measurement system, resulting in one participant-level mean value for each system. The values for skewness and kurtosis between −2 and +2 are considered acceptable in order to prove normal univariate distribution [24]. Furthermore, the Shapiro–Wilk test was used to assess whether the continuous variables were normally distributed (*p* > 0.05). Pearson’s correlation analysis was used to assess the strength of correlation between the measurements that were normally distributed, while Spearman’s rank coefficient was used for variables that were not normally distributed (*p* < 0.05 in the Shapiro–Wilk test). Correlation coefficient values are considered poor when values were below 0.20, fair for values between 0.21 and 0.50, moderate from 0.51 to 0.70, very strong from 0.71 to 0.90, and almost perfect from 0.91 to 1.00 [25]. Normally distributed descriptive statistics were compared using paired *t*-test, while non-normally distributed variables were compared using the Wilcoxon matched-pairs signed rank test. Profile plots were created using general linear model (GLM) for repeated measures ANOVA and plotting means for each gait parameter.

Reliability was calculated using ICCs with 95% confidence intervals and agreement between the two methods using profile plots. A two-way, mixed-model, average-measure ICC model was used to assess the absolute agreement in these measurements. Considering that ICC is a value between 0 and 1, values less than 0.5, between 0.5 and 0.75, between 0.75 and 0.90, and greater than 0.90 are considered indicative of poor, moderate, good, and excellent reliability, respectively [26]. An F-test from the underlying analysis of variance (ANOVA) table was used to test whether the ICC equals zero. Bland–Altman plots were used to assess whether differences between two methods were strongly associated with mean values [27]. For each variable, the plot represents the discrepancies between values of two methods against the mean values of two methods. Limits of agreement were computed as the mean difference ±1.96 SD, showing the range of discrepancies for 95% of the participants.

All analyses were conducted with IBM SPSS Statistics (v.31.0) Testing was performed two-sided, and statistical significance was defined as *p* < 0.05. To account for multiple comparisons across the four gait parameters, a Bonferroni correction was applied separately within each family of analyses (paired *t*-tests, correlation analyses, and ICC analyses), with four gait parameters included in each family. The adjusted significance threshold was therefore *p* < 0.013 (0.05/4) for each analysis family.

## 3. Results

### 3.1. Descriptive Statistics

Participants (N = 53) were age 37.9 (±16.7) and mostly female (64%) and White (75%) with a BMI of 28.2 kg/m^2^ (±5.9). Table 1 shows descriptive statistics for demographic variables.

Table 2 shows descriptive statistics for gait variables. Based on the values for skewness and kurtosis, all gait parameters, except for stride length with Smart Socks, had values for skewness and kurtosis between −2 and +2, indicating normal distribution. Sensor stride length and step count with Smart Socks did not show normality based on the Shapiro–Wilk test (*p* < 0.05).

### 3.2. Comparative Analysis

Table 3 shows descriptive values and comparative analysis between gait parameters.

Negative mean difference values indicate that the sensor measures were lower than the Smart Socks measures. Both paired *t*-test and Wilcoxon matched-pairs signed-rank test showed that significant differences were detected for four gait parameters, even after the Bonferroni correction (*p* < or ≤ 0.013). Pearson’s correlation and Spearman’s rank coefficients showed significant correlation between sensors and Smart Socks on three measures: step count, gait speed, and duration, whereas no significant correlation was found for stride length.

**Figure 1:**
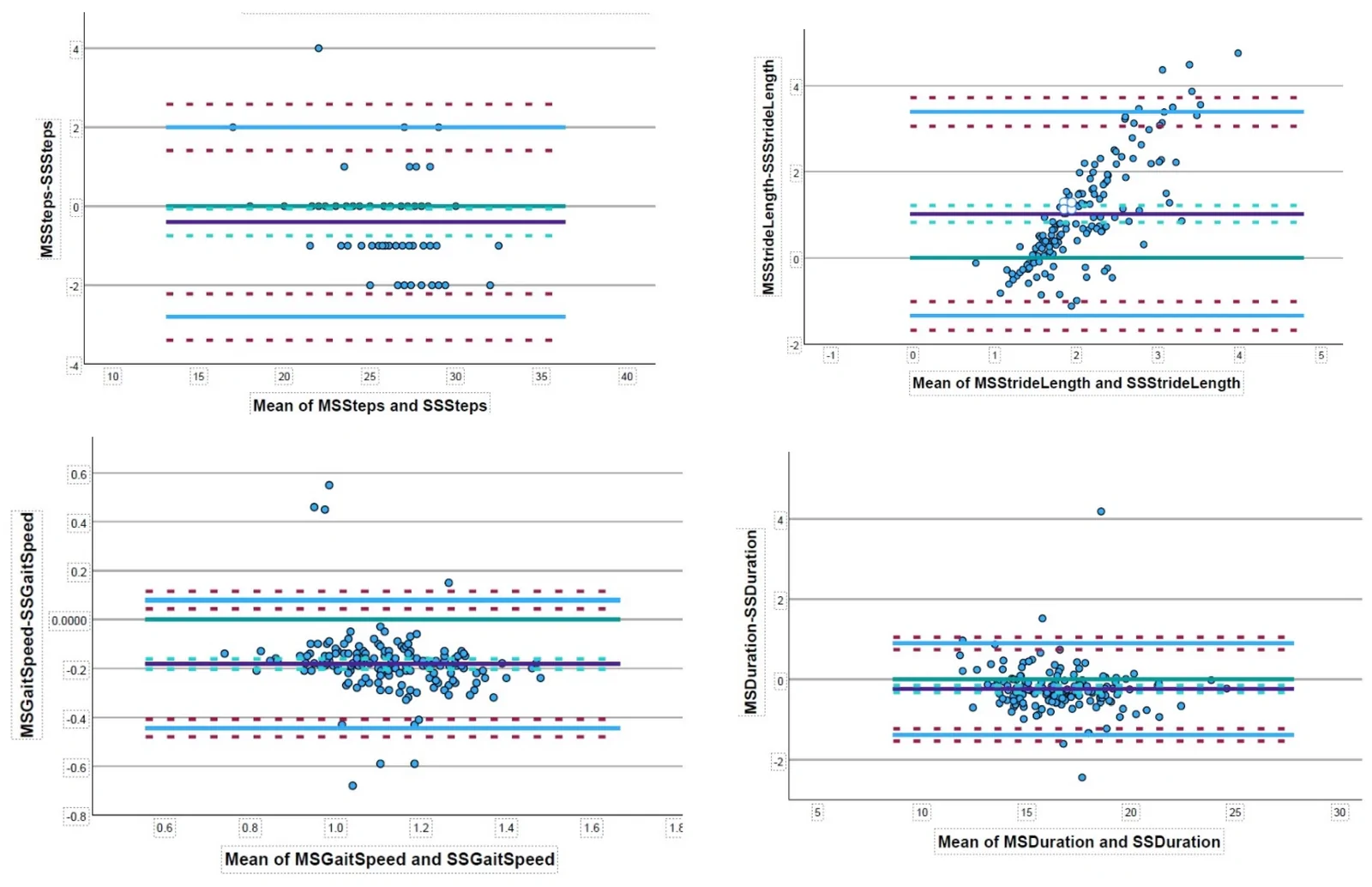
Bland–Altman plots for step count, stride length, gait speed, and duration.

Bland–Altman plots (Figure 1) based on the Bland–Altman results (Table 4) show the systematic differences and random variability between the methods, where the Y-axis shows the differences between paired measurements, while the X-axis represents the average of these measurements.

A linear regression of mean difference (as outcome) on mean (as independent variable) found no proportion bias for any of the four measures. Bland–Altman analysis estimated the mean bias and 95% limits of agreement for each gait parameter. The corresponding confidence intervals are reported in Table 4, and the plots were generated from the same participant-level differences.

### 3.3. Reliability

As shown in Table 5, the ICC values were considered excellent for step count and duration (ICC values > 0.90). The ICC value was considered good for gait speed (ICC = 0.52). Stride length demonstrated poor agreement between the two measurement systems (ICC = 0.30, 95% CI −0.185 to 0.606). The wide confidence interval, which included zero, indicated considerable uncertainty in the ICC estimate and does not support strong between-method reliability. ICC values were statistically significant based on F-test for 4 parameters even after the Bonferroni correction (all *p* < 0.013).

## 4. Discussion

The purpose of this study was to determine the inter-method reliability of Palarum PUP^®^ Smart Socks as compared to research-grade mobility sensors. Smart Socks were found to be reliable when compared to research-grade mobility sensors on step count, gait speed, and duration. While stride length revealed poor reliability, the value was statistically significant.

A variety of wearable devices are available for tracking step count and can be worn at different body locations, including the wrist, waist, thigh, and ankle. In a systematic review and meta-analysis of studies evaluating wearable reliability during walking in healthy adults, step count demonstrated excellent reliability across device types and wear locations [16]. However, the accuracy and reliability of step count measurement in hospitalized populations remains inconsistent among commercial wearables, largely due to wear location and reduced gait speed [12]. Among hospitalized adults, ankle-mounted devices have demonstrated the highest reliability for tracking step count. In contrast, thigh-worn devices have shown poor validity in older hospitalized patients, with additional measurement error observed when the device was worn on an affected limb, such as following stroke or hip surgery [18,28]. Practical limitations have also been reported for several wear locations. Waist-mounted devices may interfere with medical equipment, including cardiac monitoring leads and ostomy appliances, while thigh-mounted devices can become displaced due to patient movement or body habitus. Wrist-worn devices may cause discomfort in patients with edema or skin irritation [18]. Smart Socks have demonstrated excellent reliability and may be particularly suited for hospitalized populations because of their unobtrusive wear location, patient comfort, ease of use, and compatibility with hospital fall-prevention protocols.

Overall, while similar to the average walking duration of a hospitalized patient, a 20MWT is a relatively short walking distance and may not fully capture steady-state gait performance. During short-distance walking, a substantial proportion of the trial was influenced by gait initiation and termination, including acceleration and deceleration phases. As a result, the accuracy of the spatiotemporal gait parameters may have been impacted. In addition, a limited number of gait cycles are collected over 20 m, reducing the opportunity to assess stride-to-stride variability, which can potentially diminish the reliability of gait speed and duration. Thus, a 20MWT may be less sensitive to subtle gait abnormalities or fluctuations compared with assessments performed over longer walking distances. However, a 20MWT is often adequate to measure step count because participants typically take 20 to 40 steps, depending on stride length.

Gait analysis is important, especially among hospitalized older patients, as gait parameters are health indicators and predictors of future adverse health events [29]. The gold standard is optical motion analysis systems; however, these systems are expensive and largely immobile in clinical settings [30]. Many commercial wearables also offer gait analysis in addition to step count.

Stride length showed the largest discrepancy in agreement between the two measurement systems. The sensors yielded systematically greater stride-length values than the Smart Socks, with a mean difference of approximately 0.07 and relatively narrow Bland–Altman limits of agreement (0.062–0.097). This directional difference suggests the presence of systematic rather than exclusively random measurement variation. Differences in sensor location, gait-event detection, signal processing, calibration, and the operational definition or algorithm used to derive stride length may contribute to this discrepancy. For example, differences in identifying gait-cycle boundaries or handling the initial and terminal steps of a walking trial could systematically affect estimated stride length. Importantly, because neither system should be considered an error-free reference standard, the observed difference cannot be attributed definitively to measurement error in either system. Nevertheless, the low absolute-agreement ICC (0.30) suggests that stride-length measurements obtained from the two systems should not be used interchangeably without additional validation or calibration. This may be particularly important when stride length is used to monitor longitudinal change, identify clinically meaningful gait impairment, or compare measurements across devices. We therefore interpret the findings as evidence of limited between-method agreement for stride length rather than evidence that either system provides an inaccurate measurement.

Assessing the reliability of wearable devices for estimating stride length is challenging because stride length is not directly measured but rather estimated from sensor data using algorithms. Accuracy and reliability may be influenced by sensor type, device placement, walking speed, individual gait characteristics, and population differences. For example, the reliability found in this study may differ when assessed in hospitalized adults with slower gaits and/or altered gait patterns. Furthermore, variability in validation methods and reference standards across studies complicates comparisons and limits the generalizability of findings. Despite these challenges, a systematic review and meta-analysis evaluating the validity and reliability of wearable devices for measuring gait outcomes during level walking in healthy adults reported good-to-excellent validity and reliability for stride length across a variety of wear locations, including the back, foot, and torso [16]. However, foot-mounted devices demonstrated the strongest performance, whereas back-mounted wearables showed only poor-to-moderate validity for measuring stride length [16].

Additional studies support the superiority of foot-mounted devices for assessing stride length. Excellent inter-method reliability has been reported between FeetMe^®^ insoles and the GaitRite system, which incorporates force plates and optoelectronic motion capture technology [31]. Similarly, good agreement was found between the RehaGait system (sensors positioned on the lateral aspect of each shoe) and instrumented treadmill gait analysis (a recognized standard for measuring spatiotemporal gait parameters in adults) although differences were not statistically significant across walking speeds. Collectively, these findings suggest that foot placement may be the optimal wearable location for measuring stride length.

Self-selected gait speed, or gait velocity, is a reliable and sensitive measure of mobility that correlates strongly with functional ability and balance. As a result, gait speed is frequently used as a key indicator of physical function and recovery [32]. Consistent with its clinical utility, a systematic review examining the psychometric properties of wearable technologies in post-stroke populations reported good reliability for the measurement of gait speed, with most intraclass correlation coefficients (ICCs) ranging from 0.80 to 0.90 [33]. Despite generally favorable reliability, the accuracy of wearable devices for measuring gait speed may be influenced by slower gait speeds and device placement. In a study evaluating the validity of 15 accelerometer-based devices during treadmill walking at various speeds, slow gait speed significantly affected measurement accuracy, bias, and precision [33]. Across devices, accuracy was reduced at slower walking speeds, a finding particularly relevant for clinical and hospitalized populations. Additionally, wrist- and waist-worn devices demonstrated lower accuracy than thigh- and ankle-worn devices at slow speeds, whereas thigh- and ankle-mounted devices exhibited comparable accuracy across the range of normal walking speeds [33]. These findings suggest that wear location should be carefully considered when assessing gait speed, particularly in populations with impaired mobility or reduced walking speed, making Smart Socks a reliable wearable in the hospital setting.

Wearable devices generally demonstrate higher reliability for measuring temporal gait parameters, such as stride time or duration, than for more complex spatiotemporal parameters such as stride length. Duration is derived directly from timestamps and continuous sensor recordings, making it less dependent on algorithms and individual biomechanical characteristics [16]. Although reliability may be influenced by factors such as wear compliance, activity-detection thresholds, sensor placement, and differences in algorithms used to define the beginning and end of an activity bout, duration is generally considered one of the most robust and reproducible measures obtained from wearable devices [18]. Consistent with this observation, a systematic review and meta-analysis of wearable devices used for gait analysis reported that duration demonstrated the strongest evidence for excellent reliability among gait outcomes and maintained high reliability regardless of device placement [16]. These findings suggest that temporal gait measures may be particularly well suited for assessment using wearable technologies across a range of clinical and research settings.

Gait is a complex, multi-variable characteristic with many competing factors. Of interest is a new, practical method for gait pattern recognition: metaheuristic optimization-based selection algorithms [34]. This method works by repeatedly generating combinations of gait parameters and evaluating their quality before comparing two mobility systems. While metaheuristic optimization-based algorithms do not directly evaluate agreement between two systems, they can be used to improve the models or calibration involved in gait analysis before agreement is assessed. This method has potential to more efficiently and accurately identify gait parameters specific to different settings, such as an inpatient hospital unit, rehabilitation setting, or sports performance.

The clinical relevance of between-method agreement depends not only on statistical significance but also on whether the magnitude of the bias and limits of agreement are sufficiently small for the intended application. Accordingly, the Bland–Altman findings should be interpreted in relation to clinically or technically acceptable differences for each gait parameter. Although relatively small mean differences were observed for some measures, a small average bias does not necessarily imply interchangeability because individual measurements may still differ substantially, as reflected by the limits of agreement. For gait parameters for which established clinically acceptable thresholds are unavailable, the present findings cannot establish that the two systems are clinically interchangeable. Future studies should define a priori acceptable differences based on clinical relevance, device precision, or established reference standards and determine whether the observed limits of agreement fall within those thresholds. Particular caution is warranted for stride length because the observed systematic between-method difference, together with poor absolute agreement, suggests that measurements from the two systems should not be considered interchangeable without further validation or calibration.

The generalizability of these findings to hospitalized patients should be considered cautiously. Hospitalized patients may walk more slowly and demonstrate shorter steps, greater gait variability, shuffling or asymmetric gait, frequent stopping and starting, and altered foot-contact patterns. Some may also use assistive devices or require assistance during ambulation. These characteristics could affect sensor signal patterns and the detection of gait events, potentially influencing Smart Socks estimates of step count and spatial and temporal gait parameters. Consequently, the agreement observed in the present sample may overestimate performance in patients with impaired or atypical gait. Future studies should validate Smart Socks in hospitalized populations and specifically examine performance across clinically relevant gait-speed ranges and different gait patterns.

### Strengths and Limitations

A strength of our study was the sample size, as we slightly exceeded the minimum target range based on the power calculation. While a convenience sample, there was some diversity among participants. However, the sample was mostly female and White, compromising generalizability across all adults. In addition, all participants were younger ambulatory adults able to easily complete a 20MWT, making them different from an acutely hospitalized patient, who may be older with a slower gait or altered gait pattern. While participants were provided with the same instructions and demonstration of the 20MWT, there was wide variability in participants’ turns when completing the 20MWT. The variability may have interfered with capturing true stride length. However, our findings are similar to those of other inter-method reliability studies evaluating different wearables worn at different locations. In addition, the potential influence of gait speed on between-method agreement should also be considered. Sensor-based estimation of gait parameters may vary with walking speed because changes in gait dynamics can affect gait-event detection and the estimation of spatial and temporal measures. The present study was not designed or powered to evaluate agreement separately across gait-speed categories. Therefore, the observed agreement represents overall performance across the walking speeds represented in this sample and may not generalize equally to individuals who walk substantially slower or faster. Future studies should examine measurement agreement across prespecified gait-speed categories and include populations with a broader range of mobility and gait impairment.

## 5. Conclusions

The variability introduced by measuring stride length may be irrelevant for an EM program but should be quantified if Smart Socks will be used in a rehabilitation setting. Thus, the wearable used depends on the clinical research question, the population, their gait characteristics, and the setting. Given the good-to-excellent reliability of Palarum PUP^®^ Smart Socks to measure step count, gait speed, and duration and their optimal wear location, they show promising results for ambulatory adults. However, further validation is needed in hospitalized adults who may have slower gaits and/or altered gait patterns.

## Figures and Tables

**Table 1:** Demographic characteristics (N = 53).

Characteristics	Mean (SD) N (%)
Age (Years)	37.9 (16.7) Range 18 to 78
Gender	
Male	19 (36)
Female	34 (64)
Race	
White	40 (75.5)
Other	13 (25.5)
Weight	172.94 (41.37) Range 108 to 285

Note: SD = standard deviation.

**Table 2:** Descriptive statistics of gait parameters.

Gait Parameter	System	Mean	SD	Median	Skewness	Kurtosis	Shapiro–Wilk Test (*p*-Value)
Step Count	Sensors	25.68	2.93	26.00	−0.54	0.59	0.075
	Smart Socks	26.11	3.35	27.00	−0.73	1.08	0.021
Stride Length	Sensors	0.20	0.06	0.20	0.37	−0.70	0.126
	Smart Socks	0.13	0.05	0.11	2.36	7.36	<0.001
Gait Speed	Sensors	1.04	0.14	1.02	−0.12	−0.22	0.982
	Smart Socks	1.22	0.15	1.21	−0.42	1.15	0.603
Duration	Sensors	16.37	2.11	16.27	0.46	0.10	0.298
	Smart Socks	16.61	2.15	16.61	0.24	0.14	0.953

Note: SD = standard deviation.

**Table 3:** Comparison and correlation of gait parameters.

Gait Parameter	System	Mean	Mean Difference	*p*-Value	*p*-Value	*p*-Value	
				Paired *t*-Test	Wilcoxon Rank Test	Pearson’s Correlation	Spearman’s Correlation
Step Count	Sensors	25.68	−0.43	9.013	0.010	<0.001	<0.001
	Smart Socks	26.11					
Stride Length	Sensors	0.20	0.07	<0.001	<0.001	0.433	0.620
	Smart Socks	0.13					
Gait Speed	Sensors	1.04	−0.18	<0.001	<0.001	<0.001	<0.001
	Smart Socks	1.22					
Duration	Sensors	16.37	−0.24	<0.001	<0.001	<0.001	<0.001
	Smart Socks	16.61					

Note: Wilcoxon rank test: Wilcoxon matched-pairs signed-rank test.

**Table 4:** Bland–Altman results.

Gait Parameter	System	Mean Difference	Limits of Agreement		% Within Limits of Agreement
			Lower Bound	Upper Bound	
Step Count	Sensors	−0.43	−0.774	−0.094	95
	Smart Socks				
Stride Length	Sensors	0.08	0.062	0.097	95
	Smart Socks				
Gait Speed	Sensors	−0.18	−0.214	−0.144	95
	Smart Socks				
Duration	Sensors	−0.24	−0.331	−0.150	95
	Smart Socks				

**Table 5:** Interclass correlation coefficients (ICCs) for gait parameters.

Gait Parameter	System	ICC	95% Confidence Interval		*p*-Value
			Lower	Upper	
Step Count	Sensors	0.96	0.919	0.976	<0.001
	Smart Socks				
Stride Length	Sensors	0.32	−0.185	2.070	0.005
	Smart Socks				
Gait Speed	Sensors	0.52	−0.223	4.200	<0.001
	Smart Socks				
Duration	Sensors	0.99	0.965	1.70	<0.001
	Smart Socks				

## Data Availability

Data is available from the corresponding author upon reasonable request.

## Abbreviations

The following abbreviations are used in this manuscript:

20MWT	20 meter walk test
EM	early mobilization
ICC	intraclass correlation
USC	University of South Carolina
